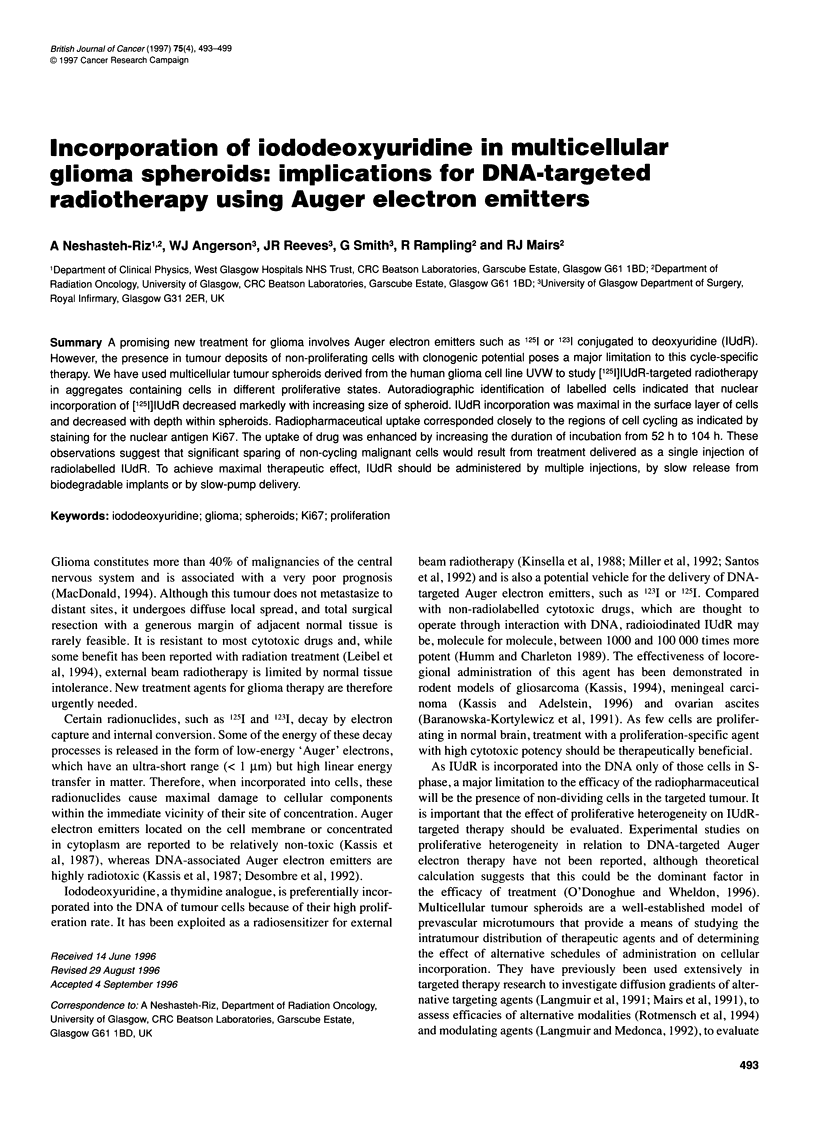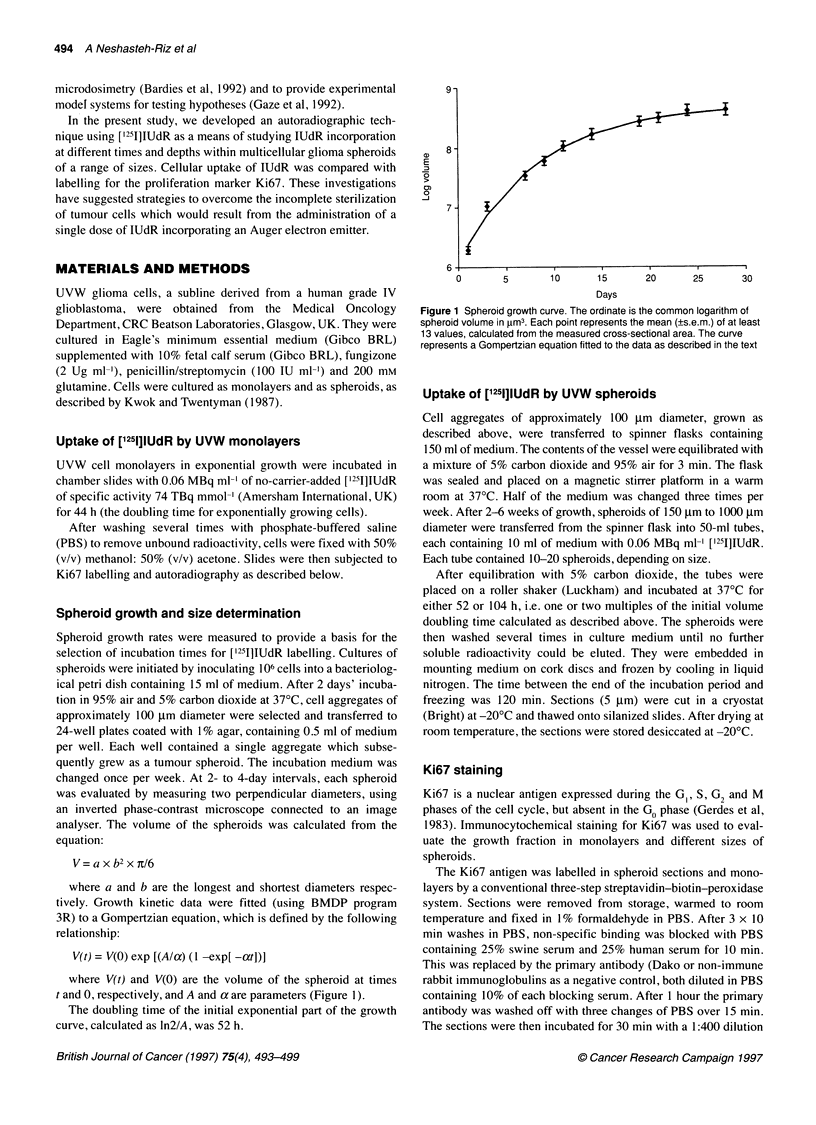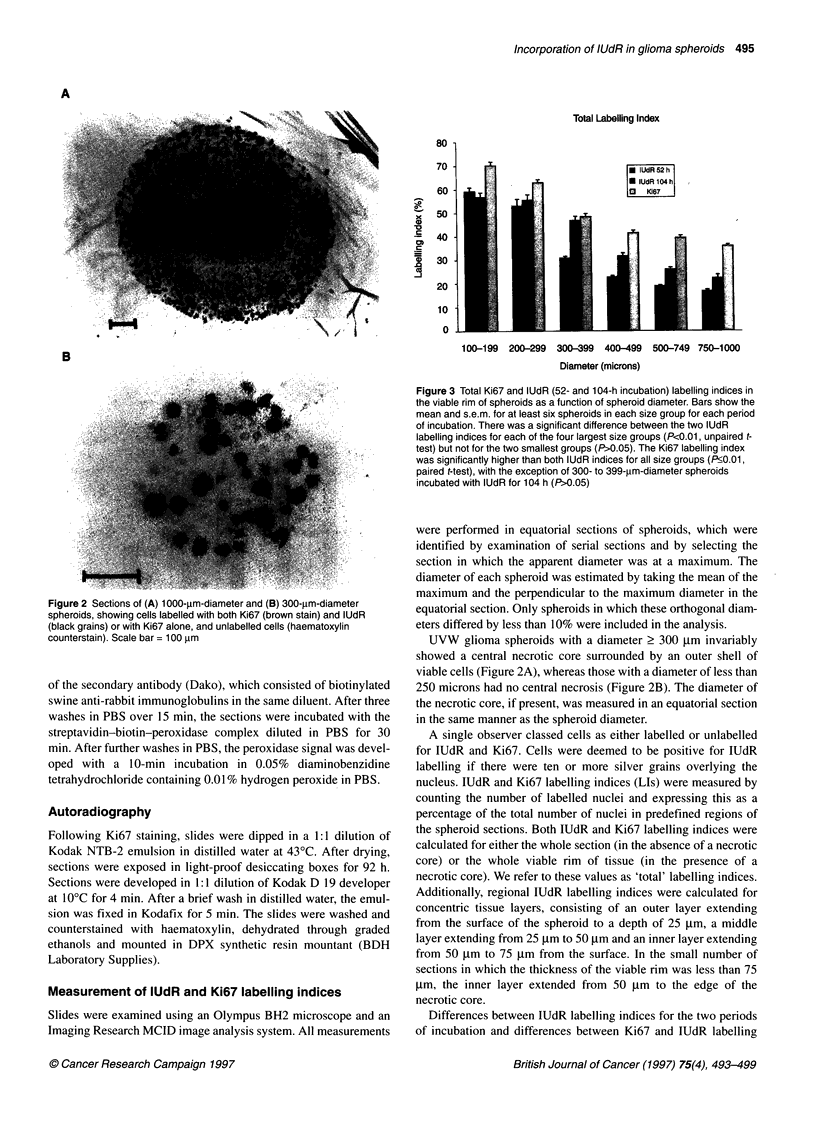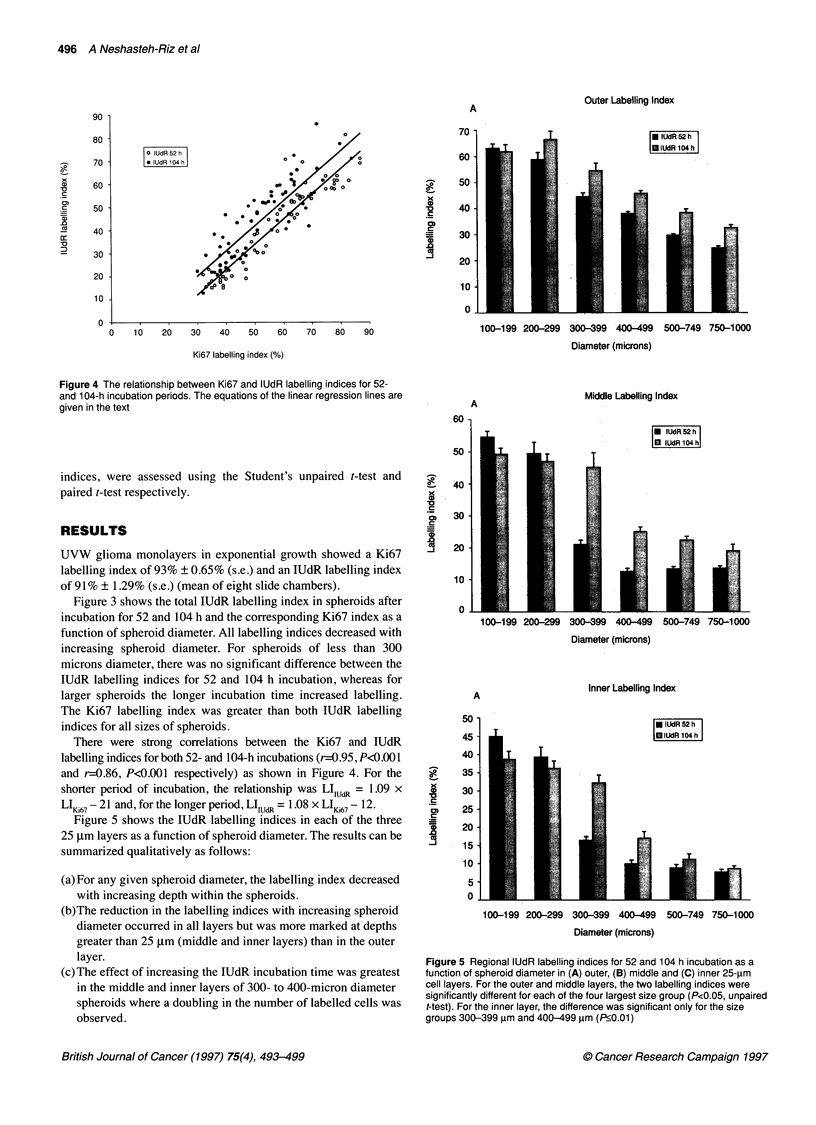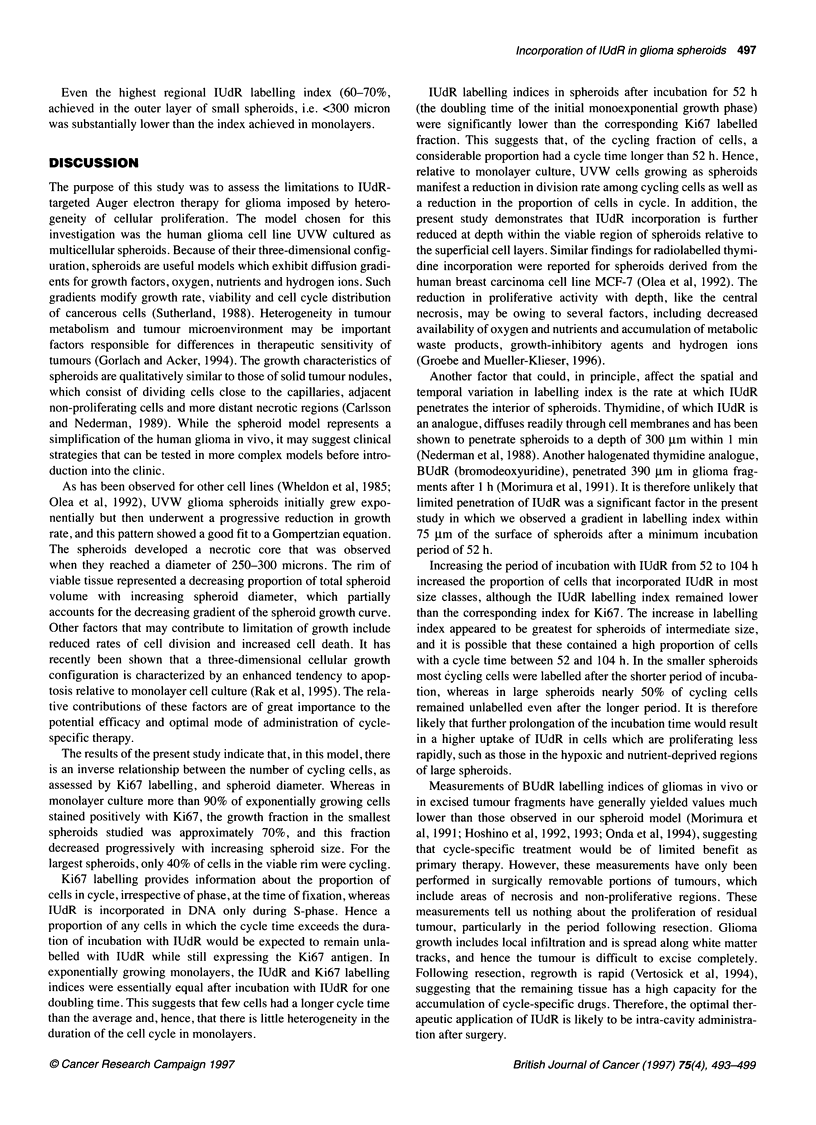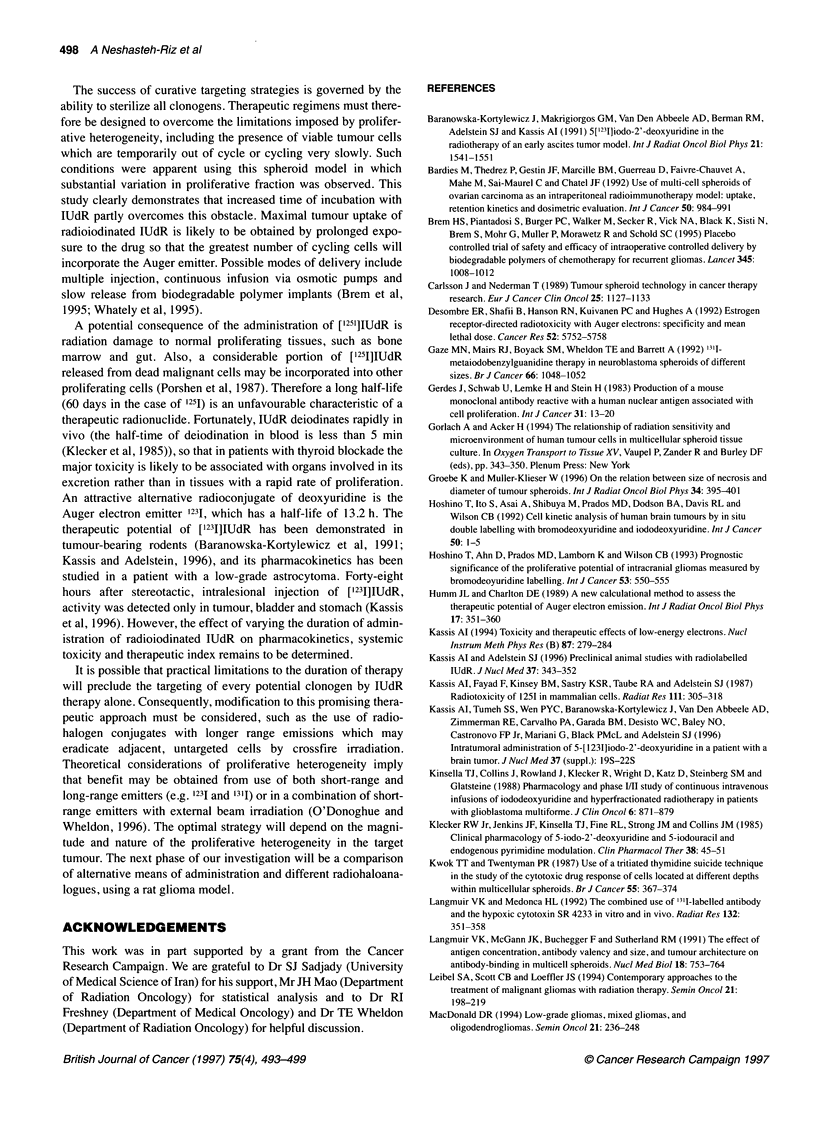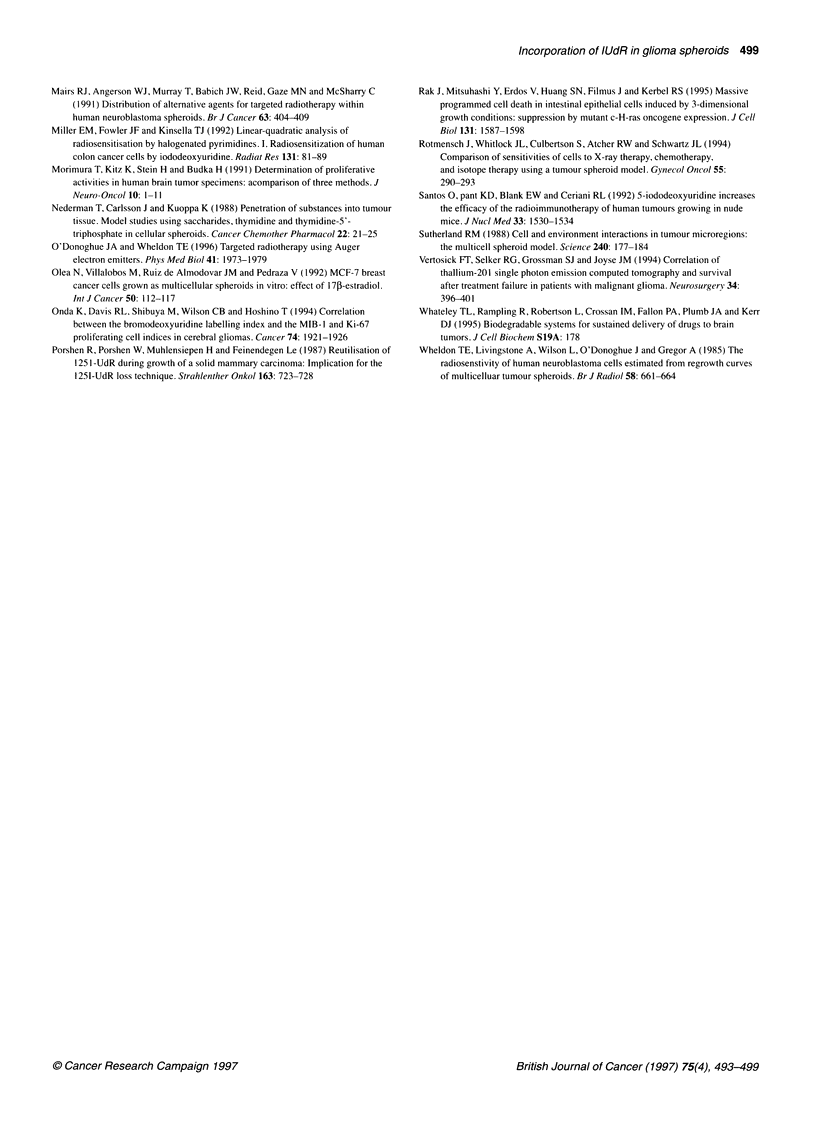# Incorporation of iododeoxyuridine in multicellular glioma spheroids: implications for DNA-targeted radiotherapy using Auger electron emitters.

**DOI:** 10.1038/bjc.1997.86

**Published:** 1997

**Authors:** A. Neshasteh-Riz, W. J. Angerson, J. R. Reeves, G. Smith, R. Rampling, R. J. Mairs

**Affiliations:** Department of Clinical Physics, West Glasgow Hospitals NHS Trust, CRC Beatson Laboratories, UK.

## Abstract

**Images:**


British Journal of Cancer (1997) 75(4), 493–499
© 1997 Cancer Research Campaign

### Incorporation of iododeoxyuridine in multicellular glioma spheroids: implications for DNA-targeted radiotherapy using Auger electron emitters

**A Neshasteh-Riz[1,2], WJ Angerson[3], JR Reeves[3], G Smith[3], R Rampling[2] and RJ Mairs[2]**

[1]Department of Clinical Physics, West Glasgow Hospitals NHS Trust, CRC Beatson Laboratories, Garscube Estate, Glasgow G61 1BD; [2]Department of Radiation Oncology, University of Glasgow, CRC Beatson Laboratories, Garscube Estate, Glasgow G61 1BD; [3]University of Glasgow Department of Surgery, Royal Infirmary, Glasgow G31 2ER, UK

**Summary** A promising new treatment for glioma involves Auger electron emitters such as $^{125}$I or $^{123}$I conjugated to deoxyuridine (IUdR). However, the presence in tumour deposits of non-proliferating cells with clonogenic potential poses a major limitation to this cycle-specific therapy. We have used multicellular tumour spheroids derived from the human glioma cell line UVW to study [$^{125}$I]IUdR-targeted radiotherapy in aggregates containing cells in different proliferative states. Autoradiographic identification of labelled cells indicated that nuclear incorporation of [$^{125}$I]IUdR decreased markedly with increasing size of spheroid. IUdR incorporation was maximal in the surface layer of cells and decreased with depth within spheroids. Radiopharmaceutical uptake corresponded closely to the regions of cell cycling as indicated by staining for the nuclear antigen Ki67. The uptake of drug was enhanced by increasing the duration of incubation from 52 h to 104 h. These observations suggest that significant sparing of non-cycling malignant cells would result from treatment delivered as a single injection of radiolabelled IUdR. To achieve maximal therapeutic effect, IUdR should be administered by multiple injections, by slow release from biodegradable implants or by slow-pump delivery.

**Keywords:** iododeoxyuridine; glioma; spheroids; Ki67; proliferation

Glioma constitutes more than 40% of malignancies of the central nervous system and is associated with a very poor prognosis (MacDonald, 1994). Although this tumour does not metastasize to distant sites, it undergoes diffuse local spread, and total surgical resection with a generous margin of adjacent normal tissue is rarely feasible. It is resistant to most cytotoxic drugs and, while some benefit has been reported with radiation treatment (Leibel et al, 1994), external beam radiotherapy is limited by normal tissue intolerance. New treatment agents for glioma therapy are therefore urgently needed.

Certain radionuclides, such as $^{125}$I and $^{123}$I, decay by electron capture and internal conversion. Some of the energy of these decay processes is released in the form of low-energy 'Auger' electrons, which have an ultra-short range (< 1 μm) but high linear energy transfer in matter. Therefore, when incorporated into cells, these radionuclides cause maximal damage to cellular components within the immediate vicinity of their site of concentration. Auger electron emitters located on the cell membrane or concentrated in cytoplasm are reported to be relatively non-toxic (Kassis et al, 1987), whereas DNA-associated Auger electron emitters are highly radiotoxic (Kassis et al, 1987; Desombre et al, 1992).

Iododeoxyuridine, a thymidine analogue, is preferentially incorporated into the DNA of tumour cells because of their high proliferation rate. It has been exploited as a radiosensitizer for external beam radiotherapy (Kinsella et al, 1988; Miller et al, 1992; Santos et al, 1992) and is also a potential vehicle for the delivery of DNA-targeted Auger electron emitters, such as $^{123}$I or $^{125}$I. Compared with non-radiolabelled cytotoxic drugs, which are thought to operate through interaction with DNA, radioiodinated IUdR may be, molecule for molecule, between 1000 and 100 000 times more potent (Humm and Charleton 1989). The effectiveness of locoregional administration of this agent has been demonstrated in rodent models of gliosarcoma (Kassis, 1994), meningeal carcinoma (Kassis and Adelstein, 1996) and ovarian ascites (Baranowska-Kortylewicz et al, 1991). As few cells are proliferating in normal brain, treatment with a proliferation-specific agent with high cytotoxic potency should be therapeutically beneficial.

As IUdR is incorporated into the DNA only of those cells in S-phase, a major limitation to the efficacy of the radiopharmaceutical will be the presence of non-dividing cells in the targeted tumour. It is important that the effect of proliferative heterogeneity on IUdR-targeted therapy should be evaluated. Experimental studies on proliferative heterogeneity in relation to DNA-targeted Auger electron therapy have not been reported, although theoretical calculation suggests that this could be the dominant factor in the efficacy of treatment (O'Donoghue and Wheldon, 1996). Multicellular tumour spheroids are a well-established model of prevascular microtumours that provide a means of studying the intratumour distribution of therapeutic agents and of determining the effect of alternative schedules of administration on cellular incorporation. They have previously been used extensively in targeted therapy research to investigate diffusion gradients of alternative targeting agents (Langmuir et al, 1991; Mairs et al, 1991), to assess efficacies of alternative modalities (Rotmensch et al, 1994) and modulating agents (Langmuir and Medonca, 1992), to evaluate

*Received 14 June 1996*
*Revised 29 August 1996*
*Accepted 4 September 1996*

*Correspondence to:* A Neshasteh-Riz, Department of Radiation Oncology, University of Glasgow, CRC Beatson Laboratories, Garscube Estate, Glasgow G61 1BD, UK

microdosimetry (Bardies et al, 1992) and to provide experimental model systems for testing hypotheses (Gaze et al, 1992).

In the present study, we developed an autoradiographic technique using [$^{125}$I]IUdR as a means of studying IUdR incorporation at different times and depths within multicellular glioma spheroids of a range of sizes. Cellular uptake of IUdR was compared with labelling for the proliferation marker Ki67. These investigations have suggested strategies to overcome the incomplete sterilization of tumour cells which would result from the administration of a single dose of IUdR incorporating an Auger electron emitter.

### MATERIALS AND METHODS

UVW glioma cells, a subline derived from a human grade IV glioblastoma, were obtained from the Medical Oncology Department, CRC Beatson Laboratories, Glasgow, UK. They were cultured in Eagle's minimum essential medium (Gibco BRL) supplemented with 10% fetal calf serum (Gibco BRL), fungizone (2 Ug ml$^{-1}$), penicillin/streptomycin (100 IU ml$^{-1}$) and 200 mM glutamine. Cells were cultured as monolayers and as spheroids, as described by Kwok and Twentyman (1987).

### Uptake of [$^{125}$I]IUdR by UVW monolayers

UVW cell monolayers in exponential growth were incubated in chamber slides with 0.06 MBq ml$^{-1}$ of no-carrier-added [$^{125}$I]IUdR of specific activity 74 TBq mmol$^{-1}$ (Amersham International, UK) for 44 h (the doubling time for exponentially growing cells).

After washing several times with phosphate-buffered saline (PBS) to remove unbound radioactivity, cells were fixed with 50% (v/v) methanol: 50% (v/v) acetone. Slides were then subjected to Ki67 labelling and autoradiography as described below.

### Spheroid growth and size determination

Spheroid growth rates were measured to provide a basis for the selection of incubation times for [$^{125}$I]IUdR labelling. Cultures of spheroids were initiated by inoculating $10^6$ cells into a bacteriological petri dish containing 15 ml of medium. After 2 days' incubation in 95% air and 5% carbon dioxide at 37°C, cell aggregates of approximately 100 μm diameter were selected and transferred to 24-well plates coated with 1% agar, containing 0.5 ml of medium per well. Each well contained a single aggregate which subsequently grew as a tumour spheroid. The incubation medium was changed once per week. At 2- to 4-day intervals, each spheroid was evaluated by measuring two perpendicular diameters, using an inverted phase-contrast microscope connected to an image analyser. The volume of the spheroids was calculated from the equation:

$$V = a \times b^2 \times \pi/6$$

where $a$ and $b$ are the longest and shortest diameters respectively. Growth kinetic data were fitted (using BMDP program 3R) to a Gompertzian equation, which is defined by the following relationship:

$$V(t) = V(0) \exp [(A/\alpha) (1 - \exp[-\alpha t])]$$

where $V(t)$ and $V(0)$ are the volume of the spheroid at times $t$ and 0, respectively, and $A$ and $\alpha$ are parameters (Figure 1).

The doubling time of the initial exponential part of the growth curve, calculated as ln2/$A$, was 52 h.

**Figure 1** Spheroid growth curve. The ordinate is the common logarithm of spheroid volume in μm$^3$. Each point represents the mean (±s.e.m.) of at least 13 values, calculated from the measured cross-sectional area. The curve represents a Gompertzian equation fitted to the data as described in the text

### Uptake of [$^{125}$I]IUdR by UVW spheroids

Cell aggregates of approximately 100 μm diameter, grown as described above, were transferred to spinner flasks containing 150 ml of medium. The contents of the vessel were equilibrated with a mixture of 5% carbon dioxide and 95% air for 3 min. The flask was sealed and placed on a magnetic stirrer platform in a warm room at 37°C. Half of the medium was changed three times per week. After 2–6 weeks of growth, spheroids of 150 μm to 1000 μm diameter were transferred from the spinner flask into 50-ml tubes, each containing 10 ml of medium with 0.06 MBq ml$^{-1}$ [$^{125}$I]IUdR. Each tube contained 10–20 spheroids, depending on size.

After equilibration with 5% carbon dioxide, the tubes were placed on a roller shaker (Luckham) and incubated at 37°C for either 52 or 104 h, i.e. one or two multiples of the initial volume doubling time calculated as described above. The spheroids were then washed several times in culture medium until no further soluble radioactivity could be eluted. They were embedded in mounting medium on cork discs and frozen by cooling in liquid nitrogen. The time between the end of the incubation period and freezing was 120 min. Sections (5 μm) were cut in a cryostat (Bright) at –20°C and thawed onto silanized slides. After drying at room temperature, the sections were stored desiccated at –20°C.

### Ki67 staining

Ki67 is a nuclear antigen expressed during the $G_1$, S, $G_2$ and M phases of the cell cycle, but absent in the $G_0$ phase (Gerdes et al, 1983). Immunocytochemical staining for Ki67 was used to evaluate the growth fraction in monolayers and different sizes of spheroids.

The Ki67 antigen was labelled in spheroid sections and monolayers by a conventional three-step streptavidin–biotin–peroxidase system. Sections were removed from storage, warmed to room temperature and fixed in 1% formaldehyde in PBS. After 3 × 10 min washes in PBS, non-specific binding was blocked with PBS containing 25% swine serum and 25% human serum for 10 min. This was replaced by the primary antibody (Dako or non-immune rabbit immunoglobulins as a negative control, both diluted in PBS containing 10% of each blocking serum. After 1 hour the primary antibody was washed off with three changes of PBS over 15 min. The sections were then incubated for 30 min with a 1:400 dilution

© Cancer Research Campaign 1997

**Figure 2** Sections of (**A**) 1000-μm-diameter and (**B**) 300-μm-diameter spheroids, showing cells labelled with both Ki67 (brown stain) and IUdR (black grains) or with Ki67 alone, and unlabelled cells (haematoxylin counterstain). Scale bar = 100 μm

**Figure 3** Total Ki67 and IUdR (52- and 104-h incubation) labelling indices in the viable rim of spheroids as a function of spheroid diameter. Bars show the mean and s.e.m. for at least six spheroids in each size group for each period of incubation. There was a significant difference between the two IUdR labelling indices for each of the four largest size groups ($P$<0.01, unpaired $t$-test) but not for the two smallest groups ($P$>0.05). The Ki67 labelling index was significantly higher than both IUdR indices for all size groups ($P$≤0.01, paired $t$-test), with the exception of 300- to 399-μm-diameter spheroids incubated with IUdR for 104 h ($P$>0.05)

of the secondary antibody (Dako), which consisted of biotinylated swine anti-rabbit immunoglobulins in the same diluent. After three washes in PBS over 15 min, the sections were incubated with the streptavidin–biotin–peroxidase complex diluted in PBS for 30 min. After further washes in PBS, the peroxidase signal was developed with a 10-min incubation in 0.05% diaminobenzidine tetrahydrochloride containing 0.01% hydrogen peroxide in PBS.

### Autoradiography

Following Ki67 staining, slides were dipped in a 1:1 dilution of Kodak NTB-2 emulsion in distilled water at 43°C. After drying, sections were exposed in light-proof desiccating boxes for 92 h. Sections were developed in 1:1 dilution of Kodak D 19 developer at 10°C for 4 min. After a brief wash in distilled water, the emulsion was fixed in Kodafix for 5 min. The slides were washed and counterstained with haematoxylin, dehydrated through graded ethanols and mounted in DPX synthetic resin mountant (BDH Laboratory Supplies).

### Measurement of IUdR and Ki67 labelling indices

Slides were examined using an Olympus BH2 microscope and an Imaging Research MCID image analysis system. All measurements were performed in equatorial sections of spheroids, which were identified by examination of serial sections and by selecting the section in which the apparent diameter was at a maximum. The diameter of each spheroid was estimated by taking the mean of the maximum and the perpendicular to the maximum diameter in the equatorial section. Only spheroids in which these orthogonal diameters differed by less than 10% were included in the analysis.

UVW glioma spheroids with a diameter ≥ 300 μm invariably showed a central necrotic core surrounded by an outer shell of viable cells (Figure 2A), whereas those with a diameter of less than 250 microns had no central necrosis (Figure 2B). The diameter of the necrotic core, if present, was measured in an equatorial section in the same manner as the spheroid diameter.

A single observer classed cells as either labelled or unlabelled for IUdR and Ki67. Cells were deemed to be positive for IUdR labelling if there were ten or more silver grains overlying the nucleus. IUdR and Ki67 labelling indices (LIs) were measured by counting the number of labelled nuclei and expressing this as a percentage of the total number of nuclei in predefined regions of the spheroid sections. Both IUdR and Ki67 labelling indices were calculated for either the whole section (in the absence of a necrotic core) or the whole viable rim of tissue (in the presence of a necrotic core). We refer to these values as 'total' labelling indices. Additionally, regional IUdR labelling indices were calculated for concentric tissue layers, consisting of an outer layer extending from the surface of the spheroid to a depth of 25 μm, a middle layer extending from 25 μm to 50 μm and an inner layer extending from 50 μm to 75 μm from the surface. In the small number of sections in which the thickness of the viable rim was less than 75 μm, the inner layer extended from 50 μm to the edge of the necrotic core.

Differences between IUdR labelling indices for the two periods of incubation and differences between Ki67 and IUdR labelling

© Cancer Research Campaign 1997

**Figure 4** The relationship between Ki67 and IUdR labelling indices for 52- and 104-h incubation periods. The equations of the linear regression lines are given in the text indices, were assessed using the Student's unpaired *t*-test and paired *t*-test respectively.

### RESULTS

UVW glioma monolayers in exponential growth showed a Ki67 labelling index of 93% ± 0.65% (s.e.) and an IUdR labelling index of 91% ± 1.29% (s.e.) (mean of eight slide chambers).

Figure 3 shows the total IUdR labelling index in spheroids after incubation for 52 and 104 h and the corresponding Ki67 index as a function of spheroid diameter. All labelling indices decreased with increasing spheroid diameter. For spheroids of less than 300 microns diameter, there was no significant difference between the IUdR labelling indices for 52 and 104 h incubation, whereas for larger spheroids the longer incubation time increased labelling. The Ki67 labelling index was greater than both IUdR labelling indices for all sizes of spheroids.

There were strong correlations between the Ki67 and IUdR labelling indices for both 52- and 104-h incubations ($r$=0.95, $P$<0.001 and $r$=0.86, $P$<0.001 respectively) as shown in Figure 4. For the shorter period of incubation, the relationship was $LI_{IUdR} = 1.09 \times LI_{Ki67} - 21$ and, for the longer period, $LI_{IUdR} = 1.08 \times LI_{Ki67} - 12$.

Figure 5 shows the IUdR labelling indices in each of the three 25 μm layers as a function of spheroid diameter. The results can be summarized qualitatively as follows:

(a) For any given spheroid diameter, the labelling index decreased with increasing depth within the spheroids.
(b) The reduction in the labelling indices with increasing spheroid diameter occurred in all layers but was more marked at depths greater than 25 μm (middle and inner layers) than in the outer layer.
(c) The effect of increasing the IUdR incubation time was greatest in the middle and inner layers of 300- to 400-micron diameter spheroids where a doubling in the number of labelled cells was observed.

**Figure 5** Regional IUdR labelling indices for 52 and 104 h incubation as a function of spheroid diameter in (**A**) outer, (**B**) middle and (**C**) inner 25-μm cell layers. For the outer and middle layers, the two labelling indices were significantly different for each of the four largest size group ($P$<0.05, unpaired *t*-test). For the inner layer, the difference was significant only for the size groups 300–399 μm and 400–499 μm ($P$≤0.01)

© Cancer Research Campaign 1997

Even the highest regional IUdR labelling index (60–70%, achieved in the outer layer of small spheroids, i.e. <300 micron was substantially lower than the index achieved in monolayers.

### DISCUSSION

The purpose of this study was to assess the limitations to IUdR-targeted Auger electron therapy for glioma imposed by heterogeneity of cellular proliferation. The model chosen for this investigation was the human glioma cell line UVW cultured as multicellular spheroids. Because of their three-dimensional configuration, spheroids are useful models which exhibit diffusion gradients for growth factors, oxygen, nutrients and hydrogen ions. Such gradients modify growth rate, viability and cell cycle distribution of cancerous cells (Sutherland, 1988). Heterogeneity in tumour metabolism and tumour microenvironment may be important factors responsible for differences in therapeutic sensitivity of tumours (Gorlach and Acker, 1994). The growth characteristics of spheroids are qualitatively similar to those of solid tumour nodules, which consist of dividing cells close to the capillaries, adjacent non-proliferating cells and more distant necrotic regions (Carlsson and Nederman, 1989). While the spheroid model represents a simplification of the human glioma in vivo, it may suggest clinical strategies that can be tested in more complex models before introduction into the clinic.

As has been observed for other cell lines (Wheldon et al, 1985; Olea et al, 1992), UVW glioma spheroids initially grew exponentially but then underwent a progressive reduction in growth rate, and this pattern showed a good fit to a Gompertzian equation. The spheroids developed a necrotic core that was observed when they reached a diameter of 250–300 microns. The rim of viable tissue represented a decreasing proportion of total spheroid volume with increasing spheroid diameter, which partially accounts for the decreasing gradient of the spheroid growth curve. Other factors that may contribute to limitation of growth include reduced rates of cell division and increased cell death. It has recently been shown that a three-dimensional cellular growth configuration is characterized by an enhanced tendency to apoptosis relative to monolayer cell culture (Rak et al, 1995). The relative contributions of these factors are of great importance to the potential efficacy and optimal mode of administration of cycle-specific therapy.

The results of the present study indicate that, in this model, there is an inverse relationship between the number of cycling cells, as assessed by Ki67 labelling, and spheroid diameter. Whereas in monolayer culture more than 90% of exponentially growing cells stained positively with Ki67, the growth fraction in the smallest spheroids studied was approximately 70%, and this fraction decreased progressively with increasing spheroid size. For the largest spheroids, only 40% of cells in the viable rim were cycling.

Ki67 labelling provides information about the proportion of cells in cycle, irrespective of phase, at the time of fixation, whereas IUdR is incorporated in DNA only during S-phase. Hence a proportion of any cells in which the cycle time exceeds the duration of incubation with IUdR would be expected to remain unlabelled with IUdR while still expressing the Ki67 antigen. In exponentially growing monolayers, the IUdR and Ki67 labelling indices were essentially equal after incubation with IUdR for one doubling time. This suggests that few cells had a longer cycle time than the average and, hence, that there is little heterogeneity in the duration of the cell cycle in monolayers.

IUdR labelling indices in spheroids after incubation for 52 h (the doubling time of the initial monoexponential growth phase) were significantly lower than the corresponding Ki67 labelled fraction. This suggests that, of the cycling fraction of cells, a considerable proportion had a cycle time longer than 52 h. Hence, relative to monolayer culture, UVW cells growing as spheroids manifest a reduction in division rate among cycling cells as well as a reduction in the proportion of cells in cycle. In addition, the present study demonstrates that IUdR incorporation is further reduced at depth within the viable region of spheroids relative to the superficial cell layers. Similar findings for radiolabelled thymidine incorporation were reported for spheroids derived from the human breast carcinoma cell line MCF-7 (Olea et al, 1992). The reduction in proliferative activity with depth, like the central necrosis, may be owing to several factors, including decreased availability of oxygen and nutrients and accumulation of metabolic waste products, growth-inhibitory agents and hydrogen ions (Groebe and Mueller-Klieser, 1996).

Another factor that could, in principle, affect the spatial and temporal variation in labelling index is the rate at which IUdR penetrates the interior of spheroids. Thymidine, of which IUdR is an analogue, diffuses readily through cell membranes and has been shown to penetrate spheroids to a depth of 300 μm within 1 min (Nederman et al, 1988). Another halogenated thymidine analogue, BUdR (bromodeoxyuridine), penetrated 390 μm in glioma fragments after 1 h (Morimura et al, 1991). It is therefore unlikely that limited penetration of IUdR was a significant factor in the present study in which we observed a gradient in labelling index within 75 μm of the surface of spheroids after a minimum incubation period of 52 h.

Increasing the period of incubation with IUdR from 52 to 104 h increased the proportion of cells that incorporated IUdR in most size classes, although the IUdR labelling index remained lower than the corresponding index for Ki67. The increase in labelling index appeared to be greatest for spheroids of intermediate size, and it is possible that these contained a high proportion of cells with a cycle time between 52 and 104 h. In the smaller spheroids most cycling cells were labelled after the shorter period of incubation, whereas in large spheroids nearly 50% of cycling cells remained unlabelled even after the longer period. It is therefore likely that further prolongation of the incubation time would result in a higher uptake of IUdR in cells which are proliferating less rapidly, such as those in the hypoxic and nutrient-deprived regions of large spheroids.

Measurements of BUdR labelling indices of gliomas in vivo or in excised tumour fragments have generally yielded values much lower than those observed in our spheroid model (Morimura et al, 1991; Hoshino et al, 1992, 1993; Onda et al, 1994), suggesting that cycle-specific treatment would be of limited benefit as primary therapy. However, these measurements have only been performed in surgically removable portions of tumours, which include areas of necrosis and non-proliferative regions. These measurements tell us nothing about the proliferation of residual tumour, particularly in the period following resection. Glioma growth includes local infiltration and is spread along white matter tracks, and hence the tumour is difficult to excise completely. Following resection, regrowth is rapid (Vertosick et al, 1994), suggesting that the remaining tissue has a high capacity for the accumulation of cycle-specific drugs. Therefore, the optimal therapeutic application of IUdR is likely to be intra-cavity administration after surgery.

© Cancer Research Campaign 1997

The success of curative targeting strategies is governed by the ability to sterilize all clonogens. Therapeutic regimens must therefore be designed to overcome the limitations imposed by proliferative heterogeneity, including the presence of viable tumour cells which are temporarily out of cycle or cycling very slowly. Such conditions were apparent using this spheroid model in which substantial variation in proliferative fraction was observed. This study clearly demonstrates that increased time of incubation with IUdR partly overcomes this obstacle. Maximal tumour uptake of radioiodinated IUdR is likely to be obtained by prolonged exposure to the drug so that the greatest number of cycling cells will incorporate the Auger emitter. Possible modes of delivery include multiple injection, continuous infusion via osmotic pumps and slow release from biodegradable polymer implants (Brem et al, 1995; Whately et al, 1995).

A potential consequence of the administration of [$^{125}$I]IUdR is radiation damage to normal proliferating tissues, such as bone marrow and gut. Also, a considerable portion of [$^{125}$I]IUdR released from dead malignant cells may be incorporated into other proliferating cells (Porshen et al, 1987). Therefore a long half-life (60 days in the case of $^{125}$I) is an unfavourable characteristic of a therapeutic radionuclide. Fortunately, IUdR deiodinates rapidly in vivo (the half-time of deiodination in blood is less than 5 min (Klecker et al, 1985)), so that in patients with thyroid blockade the major toxicity is likely to be associated with organs involved in its excretion rather than in tissues with a rapid rate of proliferation. An attractive alternative radioconjugate of deoxyuridine is the Auger electron emitter $^{123}$I, which has a half-life of 13.2 h. The therapeutic potential of [$^{123}$I]IUdR has been demonstrated in tumour-bearing rodents (Baranowska-Kortylewicz et al, 1991; Kassis and Adelstein, 1996), and its pharmacokinetics has been studied in a patient with a low-grade astrocytoma. Forty-eight hours after stereotactic, intralesional injection of [$^{123}$I]IUdR, activity was detected only in tumour, bladder and stomach (Kassis et al, 1996). However, the effect of varying the duration of administration of radioiodinated IUdR on pharmacokinetics, systemic toxicity and therapeutic index remains to be determined.

It is possible that practical limitations to the duration of therapy will preclude the targeting of every potential clonogen by IUdR therapy alone. Consequently, modification to this promising therapeutic approach must be considered, such as the use of radiohalogen conjugates with longer range emissions which may eradicate adjacent, untargeted cells by crossfire irradiation. Theoretical considerations of proliferative heterogeneity imply that benefit may be obtained from use of both short-range and long-range emitters (e.g. $^{123}$I and $^{131}$I) or in a combination of short-range emitters with external beam irradiation (O'Donoghue and Wheldon, 1996). The optimal strategy will depend on the magnitude and nature of the proliferative heterogeneity in the target tumour. The next phase of our investigation will be a comparison of alternative means of administration and different radiohaloanalogues, using a rat glioma model.

### ACKNOWLEDGEMENTS

This work was in part supported by a grant from the Cancer Research Campaign. We are grateful to Dr SJ Sadjady (University of Medical Science of Iran) for his support, Mr JH Mao (Department of Radiation Oncology) for statistical analysis and to Dr RI Freshney (Department of Medical Oncology) and Dr TE Wheldon (Department of Radiation Oncology) for helpful discussion.

### REFERENCES

Baranowska-Kortylewicz J, Makrigiorgos GM, Van Den Abbeele AD, Berman RM, Adelstein SJ and Kassis AI (1991) 5[$^{123}$I]iodo-2'-deoxyuridine in the radiotherapy of an early ascites tumor model. *Int J Radiat Oncol Biol Phys* **21**: 1541–1551

Bardies M, Thedrez P, Gestin JF, Marcille BM, Guerreau D, Faivre-Chauvet A, Mahe M, Sai-Maurel C and Chatel JF (1992) Use of multi-cell spheroids of ovarian carcinoma as an intraperitoneal radioimmunotherapy model: uptake, retention kinetics and dosimetric evaluation. *Int J Cancer* **50**: 984–991

Brem HS, Piantadosi S, Burger PC, Walker M, Secker R, Vick NA, Black K, Sisti N, Brem S, Mohr G, Muller P, Morawetz R and Schold SC (1995) Placebo controlled trial of safety and efficacy of intraoperative controlled delivery by biodegradable polymers of chemotherapy for recurrent gliomas. *Lancet* **345**: 1008–1012

Carlsson J and Nederman T (1989) Tumour spheroid technology in cancer therapy research. *Eur J Cancer Clin Oncol* **25**: 1127–1133

Desombre ER, Shafii B, Hanson RN, Kuivanen PC and Hughes A (1992) Estrogen receptor-directed radiotoxicity with Auger electrons: specificity and mean lethal dose. *Cancer Res* **52**: 5752–5758

Gaze MN, Mairs RJ, Boyack SM, Wheldon TE and Barrett A (1992) $^{131}$I-metaiodobenzylguanidine therapy in neuroblastoma spheroids of different sizes. *Br J Cancer* **66**: 1048–1052

Gerdes J, Schwab U, Lemke H and Stein H (1983) Production of a mouse monoclonal antibody reactive with a human nuclear antigen associated with cell proliferation. *Int J Cancer* **31**: 13–20

Gorlach A and Acker H (1994) The relationship of radiation sensitivity and microenvironment of human tumour cells in multicellular spheroid tissue culture. In *Oxygen Transport to Tissue XV*, Vaupel P, Zander R and Burley DF (eds), pp. 343–350. Plenum Press: New York

Groebe K and Muller-Klieser W (1996) On the relation between size of necrosis and diameter of tumour spheroids. *Int J Radiat Oncol Biol Phys* **34**: 395–401

Hoshino T, Ito S, Asai A, Shibuya M, Prados MD, Dodson BA, Davis RL and Wilson CB (1992) Cell kinetic analysis of human brain tumours by in situ double labelling with bromodeoxyuridine and iododeoxyuridine. *Int J Cancer* **50**: 1–5

Hoshino T, Ahn D, Prados MD, Lamborn K and Wilson CB (1993) Prognostic significance of the proliferative potential of intracranial gliomas measured by bromodeoyuridine labelling. *Int J Cancer* **53**: 550–555

Humm JL and Charlton DE (1989) A new calculational method to assess the therapeutic potential of Auger electron emission. *Int J Radiat Oncol Biol Phys* **17**: 351–360

Kassis AI (1994) Toxicity and therapeutic effects of low-energy electrons. *Nucl Instrum Meth Phys Res* (B) **87**: 279–284

Kassis AI and Adelstein SJ (1996) Preclinical animal studies with radiolabelled IUdR. *J Nucl Med* **37**: 343–352

Kassis AI, Fayad F, Kinsey BM, Sastry KSR, Taube RA and Adelstein SJ (1987) Radiotoxicity of 125I in mammalian cells. *Radiat Res* **111**: 305–318

Kassis AI, Tumeh SS, Wen PYC, Baranowska-Kortylewicz J, Van Den Abbeele AD, Zimmerman RE, Carvalho PA, Garada BM, Desisto WC, Baley NO, Castronovo FP Jr, Mariani G, Black PMcL and Adelstein SJ (1996) Intratumoral administration of 5-[123I]iodo-2'-deoxyuridine in a patient with a brain tumor. *J Nucl Med* **37** (suppl.): 19S–22S

Kinsella TJ, Collins J, Rowland J, Klecker R, Wright D, Katz D, Steinberg SM and Glatsteine (1988) Pharmacology and phase I/II study of continuous intravenous infusions of iododeoxyuridine and hyperfractionated radiotherapy in patients with glioblastoma multiforme. *J Clin Oncol* **6**: 871–879

Klecker RW Jr, Jenkins JF, Kinsella TJ, Fine RL, Strong JM and Collins JM (1985) Clinical pharmacology of 5-iodo-2'-deoxyuridine and 5-iodouracil and endogenous pyrimidine modulation. *Clin Pharmacol Ther* **38**: 45–51

Kwok TT and Twentyman PR (1987) Use of a tritiated thymidine suicide technique in the study of the cytotoxic drug response of cells located at different depths within multicellular spheroids. *Br J Cancer* **55**: 367–374

Langmuir VK and Medonca HL (1992) The combined use of $^{131}$I-labelled antibody and the hypoxic cytotoxin SR 4233 in vitro and in vivo. *Radiat Res* **132**: 351–358

Langmuir VK, McGann JK, Buchegger F and Sutherland RM (1991) The effect of antigen concentration, antibody valency and size, and tumour architecture on antibody-binding in multicell spheroids. *Nucl Med Biol* **18**: 753–764

Leibel SA, Scott CB and Loeffler JS (1994) Contemporary approaches to the treatment of malignant gliomas with radiation therapy. *Semin Oncol* **21**: 198–219

MacDonald DR (1994) Low-grade gliomas, mixed gliomas, and oligodendrogliomas. *Semin Oncol* **21**: 236–248

© Cancer Research Campaign 1997

Mairs RJ, Angerson WJ, Murray T, Babich JW, Reid, Gaze MN and McSharry C (1991) Distribution of alternative agents for targeted radiotherapy within human neuroblastoma spheroids. *Br J Cancer* **63**: 404–409

Miller EM, Fowler JF and Kinsella TJ (1992) Linear-quadratic analysis of radiosensitisation by halogenated pyrimidines. I. Radiosensitization of human colon cancer cells by iododeoxyuridine. *Radiat Res* **131**: 81–89

Morimura T, Kitz K, Stein H and Budka H (1991) Determination of proliferative activities in human brain tumor specimens: acomparison of three methods. *J Neuro-Oncol* **10**: 1–11

Nederman T, Carlsson J and Kuoppa K (1988) Penetration of substances into tumour tissue. Model studies using saccharides, thymidine and thymidine-5'-triphosphate in cellular spheroids. *Cancer Chemother Pharmacol* **22**: 21–25

O'Donoghue JA and Wheldon TE (1996) Targeted radiotherapy using Auger electron emitters. *Phys Med Biol* **41**: 1973–1979

Olea N, Villalobos M, Ruiz de Almodovar JM and Pedraza V (1992) MCF-7 breast cancer cells grown as multicellular spheroids in vitro: effect of 17β-estradiol. *Int J Cancer* **50**: 112–117

Onda K, Davis RL, Shibuya M, Wilson CB and Hoshino T (1994) Correlation between the bromodeoxyuridine labelling index and the MIB-1 and Ki-67 proliferating cell indices in cerebral gliomas. *Cancer* **74**: 1921–1926

Porshen R, Porshen W, Muhlensiepen H and Feinendegen Le (1987) Reutilisation of 1251-UdR during growth of a solid mammary carcinoma: Implication for the 125I-UdR loss technique. *Strahlenther Onkol* **163**: 723–728

Rak J, Mitsuhashi Y, Erdos V, Huang SN, Filmus J and Kerbel RS (1995) Massive programmed cell death in intestinal epithelial cells induced by 3-dimensional growth conditions: suppression by mutant c-H-ras oncogene expression. *J Cell Biol* **131**: 1587–1598

Rotmensch J, Whitlock JL, Culbertson S, Atcher RW and Schwartz JL (1994) Comparison of sensitivities of cells to X-ray therapy, chemotherapy, and isotope therapy using a tumour spheroid model. *Gynecol Oncol* **55**: 290–293

Santos O, pant KD, Blank EW and Ceriani RL (1992) 5-iododeoxyuridine increases the efficacy of the radioimmunotherapy of human tumours growing in nude mice. *J Nucl Med* **33**: 1530–1534

Sutherland RM (1988) Cell and environment interactions in tumour microregions: the multicell spheroid model. *Science* **240**: 177–184

Vertosick FT, Selker RG, Grossman SJ and Joyse JM (1994) Correlation of thallium-201 single photon emission computed tomography and survival after treatment failure in patients with malignant glioma. *Neurosurgery* **34**: 396–401

Whateley TL, Rampling R, Robertson L, Crossan IM, Fallon PA, Plumb JA and Kerr DJ (1995) Biodegradable systems for sustained delivery of drugs to brain tumors. *J Cell Biochem* **S19A**: 178

Wheldon TE, Livingstone A, Wilson L, O'Donoghue J and Gregor A (1985) The radiosenstivity of human neuroblastoma cells estimated from regrowth curves of multicelluar tumour spheroids. *Br J Radiol* **58**: 661–664

© Cancer Research Campaign 1997